# CrossFit® open performance is affected by the nature of past competition experiences

**DOI:** 10.1186/s13102-022-00434-0

**Published:** 2022-03-24

**Authors:** Gerald T. Mangine, Jacob M. McDougle

**Affiliations:** 1 Exercise Science and Sport Management, Kennesaw State University, 520 Parliament Garden Way NW, Kennesaw, GA 30144 USA; 2 Kinesiology, University of Connecticut, Storrs, CT USA

**Keywords:** Team, Games, Athlete, High-intensity functional training, HIFT

## Abstract

**Purpose:**

To examine the relationships between past competition performances and 2020 CrossFit® Open (CFO) performance.

**Methods:**

A random selection from the top one thousand athletes (*n* = 220, 28.5 ± 4.4 years, 178 ± 7 cm, 87.5 ± 10.2 kg) were selected for this study. Overall and weekly performances (including ranks and scores) of the 2020 CFO, as well as overall ranks from all previous CFO, regional, and Games™ competitions in which they competed, were recorded from their publicly available online profile. The highest, lowest, average, and standard deviation (SD) of past rankings, as well as participation statistics (i.e., years since first appearance, total and consecutive appearances, and participation rate), were calculated for each competition stage. Relationships were then assessed between 2020 CFO performance and all past competition experience variables by calculating Kendall’s tau (τ) correlation coefficients and Bayes factors (BF_10_).

**Results:**

Overall and weekly ranking of the 2020 CFO was *extremely* favored (*p* < 0.001, BF_10_ > 100) to be related to the athlete’s highest previous CFO rank (τ = 0.26–0.39) and individual regional appearances (τ = − 0.26 to − 0.34), as well as individual Games™ appearances (overall and for weeks 1, 3, and 4; τ = − 0.20 to − 0.22, *p* < 0.001, BF_10_ > 100). Evidence for all other significant relationships ranged from *moderate* to *very strong* (*p* < 0.05, BF_10_ = 3–100) and varied among specific 2020 CFO workouts. Few associations were noted for team competition experience, and these were generally limited to Games™ appearances (τ = − 0.12 to − 0.18, *p* < 0.05, BF_10_ = 3.3–100).

**Conclusions:**

Although specific relationships were found between 2020 CFO performance and individual appearances at regional and Games™ competitions, the most consistent relationships were seen with participation and ranking in past CFO competitions.

## Background

Thorough understanding of the demands of a sport and the primary factors that underpin success allows coaches, athletes, and sports scientists to develop and implement effective training plans. Strength and conditioning professionals may identify relevant energy systems, common movement patterns, and injury-health risks, which enables them to develop a series of tests to quantify the athlete’s aptitude in associated physiological indicators of performance [1, 2]. In contrast, actual skill in a sport appears to be commonly quantified by either the athlete’s on-field performance or simply implied by their competitive level and years of experience [3, 4]. Unlike most traditional sports, however, the broad demands of CrossFit® (CF) make it difficult to classify and identify the factors related to success. Consistent with its mission [5], CF competitions typically involve multiple events that variably emphasize proficiency in several areas of fitness; the Level 1 Training Guide states that CF training targets 10 specific areas of fitness. Accordingly, studies have noted relationships between CF performance and multiple areas of fitness, including body composition, muscle architecture, strength, anaerobic threshold and power, critical speed and power, repeated sprinting ability, and aerobic capacity [6–12]. This differs from more traditional sports where modality and relevant physiology are typically fixed during gameplay and across levels of competition. The lack of similarities between CF and more traditional sports also limits the insight their more established scientific records might provide. With limited evidence and no consensus as to which traits are most important, CF athletes must decide on whether to specialize or train for proficiency in everything. This decision is further complicated by their current competitive level, experience, and competence within each area.

Athletes are classified by a combination of details about their demographic, biometrics, physiological performance indicators, and athletic experiences [1, 3, 4, 13]. Of these, experience may be the broadest and most subjective, yet it is simply and commonly quantified by years of participation in a specific sport or activity. The utility of this appears to be based on several assumptions. As an athlete progresses in age and competitive level, game play in the sport usually becomes more sophisticated, which alters demands and determinants of success. An older or more experienced athlete might be assumed to already have proficient relevant physiological adaptations, acquired essential sport-specific skills, and thus, have more specific training requirements than their novice counterparts [14, 15]. The veracity of these assumptions depends on the composition and quality of the athlete’s relevant experiences, which differ amongst individuals. An athlete may play a sport for several years and still lack the physical or tactical skills commonly associated with a high level of play and success. Conversely, another athlete may enjoy success after a limited amount of sport-specific experiences because they can draw upon skills learned from other sporting (and non-sporting) environments, may have developed under superior coaching, and/or they faced (or trained with) athletes who possess more experience, talent, or skill than themselves [16, 17]. To this end, Gulbin et al. [18] identified eight unique developmental trajectories of elite athletes, and these predominantly involve multiple transitions between higher and lower levels of competition. Ability is challenged when an athlete is exposed to a more competitive environment, and this exposure helps stimulate improvements in tactical and technical skills. Learned skills may also be refined when the athlete returns to a less competitive environment which further assists the athlete’s ascension to higher levels of play. Years of involvement (with a particular sport) cannot encapsulate such developments, and consequently, limits the practical relevance of any study-related recommendations when it is used as the sole descriptor of experience.

Of interest to the present investigation, a pair of studies have cited CF experience as being an important predictor of performance [6, 9]. Bellar and colleagues [9] recruited men who either had no CF experience (*n* = 11) or at least one year of CF experience (*n* = 21) including local competition experience; though one participant possessed Games™ experience and four had regional experience. The authors found that years of CF experience, rather than aerobic or anaerobic power, was a better and more consistent predictor of performance in two novel, laboratory-based workouts. Mangine et al. [6] expanded on this by examining relationships between 2018 CrossFit® Open (CFO) performance and several descriptors of CF training and competition experience. Half of the men and women in that study (*n* = 8) possessed regional and Games™ experience as members of teams (one also had experience as an individual competitor), whereas the others had never progressed beyond the CFO. Here, the nature of the athletes’ competitive experiences (i.e., individual vs. team experience, CFO vs. regionals vs. Games™) ranged in how well they could explain variance in each workout (*r*^2^ = 0.04–0.59). Despite being limited to two smaller convenience samples; CF experience is clearly a worthy consideration for performance that should be quantified by more information than just years of participation.

Since the first Games™ competition in 2007, there have been several modifications to the competition’s structure. Most notably, preliminary rounds (i.e., regionals and CFO) were added in 2011, the regional round was removed in 2018 and replaced by quarter- and semi-finals in 2021, and the duration of the CFO was reduced from 5 to 3 weeks in 2021 [19]. Though these changes are likely representative of the sport’s growth in popularity and an effort to promote fair competition, they have unavoidably altered the competition itself and the possibility for athletes to gain certain experiences. The importance of some those potential experiences have yet to be fully understood. Experience is considered an asset for many sports [13, 15, 18, 20], and in CF, it may help explain why a more experienced athlete may outperform a less experienced, but more physically and physiologically gifted athlete. Familiarity with various exercises and workout structures, effective transitions between exercises and modalities, self-awareness, and planning skills may all improve with experience [21–24]. Before these can be verified to occur following CF training, however, the relevance of different types of experience must first be established. Therefore, the purpose of this study was to examine the relationships between past CF competition performances and 2020 CFO performance in large group of successful athletes.

## Methods

### Study design

To examine the relationships between past competition experiences and 2020 CFO performance, all study-related data was collected from a publicly available online leaderboard [25]. The leaderboard maintains all performance data from official CF competitions ranging from 2007 to present. An athlete’s competitive history at any of these events may be accessed via the leaderboard and their linked online profile. For the present study, the score and final rank earned for each workout of the 2020 CFO, as well as competitive history (i.e., official ranking at all past CF events), was recorded for a random selection of registered athletes. Competition history data was collected from 2012 to present due to 2011 being the first year of the CFO and regional rounds, and 2011 CFO data not being available on the online leaderboard. Data was then treated to enable pairwise relationship analysis between 2020 CFO performance and past CF competition experience variables.

### Participants

Using previously observed relationships [6], a priori analysis with G*Power (v. 3.1.9.7, Heinrich-Heine-Universität, Germany) indicated that a minimum sample size of 208 were needed for the present study. Consequently, 220 athletes (n = 220, 28.5 ± 4.4 years, 178 ± 7 cm, 87.5 ± 10.2 kg), ranked between 1st and 1000th place (520 ± 281) in the men’s division of the 2020 CFO competition, were randomly selected for this study. Although no stratification procedures were employed, the random selection process was limited to athletes who ranked within the top 1000 places to improve the likelihood that athletes with regional and Games™ experience would be sufficiently represented in the sample. All athletes possessed a profile on the CrossFit Games™ website [25] and were randomly selected using the random number generator and index functions of computer software (Microsoft Excel v. 365, Microsoft Corporation, Redmond, VA, USA). For verification purposes, only athletes who submitted a video recording of themselves completing each 2020 CFO workout along with their respective scores were considered. The study was conducted in accordance with the Declaration of Helsinki. Since all data were pre-existing and publicly available, the University’s Institutional Review Board classified this study as exempt (#16-215).

### 2020 CFO competition

The 2020 CFO competition consisted of 5 unique workouts (W1–W5) released over 5 weeks. Competition rules allotted four days each week (Thursday at 1700 h Pacific Daylight Time [PDT] to Monday at 1700 h PDT) for competitors to complete the assigned workout and submit their best score to their online profile [25]. The athletes were able to complete all workouts at their normal training facility, provided their submission was supported by an official competition judge’s signature (verifying that they observed and certified the complete attempt) or a video recording of the attempt. Competition representatives then reviewed submissions for accuracy before updating their status as being official. The performances retained for this study represent the athletes’ official and best attempt on each 2020 CFO workout.

Except for W2 (scored as repetitions completed), all workouts were officially scored as time-to-completion (TTC), or repetitions completed if they did not complete all assigned work within each workout’s respective time limit. Since some competitors within the present sample did not complete W3 or W4 within the assigned time limit, performance in these workouts was quantified in two different ways. Consequently, it was necessary to consolidate these scores into a single value (i.e., rate of repetitions completed per minute of exercise; reps * min^−1^). Descriptions of 2020 CFO workouts (W1–W5) and athlete performances are presented in Table 1.

**Table 1:** 2020 CFO workout descriptions and competitor performances

Workout design		Official score	Competitor ranks and investigation scores
W1	*10 rounds alternating:*	TTC or repetitions completed in 15 min	1001st ± 904th (5th–5088th) 643 ± 54 s (499–771 s)
	8 × ground to overhead (95/65 lbs.)		
	10 × Bar-facing burpees		
W2	*20-min AMRAP*	Repetitions completed	846th ± 760th (7th—5322nd) 849 ± 57 repetitions (709–1015 repetitions)
	4 × dumbbell thrusters (50/35 lbs.)		
	6 × toes-to-bar		
	24 × double-Unders		
W3	*3 Rounds of 21-15-9 repetitions*	TTC or repetitions completed in 9 min	868th ± 807th (4th–5616th) 18.0 ± 2.3 reps/min (13.6–26.1 reps/min)
	Deadlifts (225/155 lbs.)		
	Handstand push-ups		
	*Immediately into 3 Rounds of 21-15-9 repetitions*		
	Deadlifts (315/205 lbs.)		
	50-ft handstand walk		
W4	*20 min to complete work in the following order:*	TTC or repetitions completed in 20 min	935th ± 876th (11th–6653rd) 12.0 ± 0.7 reps/min (10.0–15.1 reps/min)
	30 × box jumps (24/20 in) → 15 × clean and Jerks (95/65 lbs.)		
	30 × box jumps (24/20 in) → 15 × clean and Jerks (135/85 lbs.)		
	30 × Box jumps (24/20 in) → 10 × clean and Jerks (185/115 lbs.)		
	30 × Single-leg squats → 10 × clean and Jerks (225/145 lbs.)		
	30 × Single-leg squats → 5 × clean and Jerks (275/175 lbs.)		
	30 × Single-leg squats → 5 × clean and Jerks (315/205 lbs.)		
W5	*20 min to complete the following work in any order:*	TTC or repetitions completed in 20 min	776th ± 678th (1st–4345th) 757 ± 59 s (594–933 s)
	40 muscle-ups		
	80-cal row		
	120 wall ball shots (20-lbs. to 10-ft/14-lbs. to 9ft)		

### Competition experience

Participation in previous CFO (or higher) competitions and final ranking were recorded from each athletes’ online profile [26]. The recorded information included the competition title (CFO, Regionals, or the Games™), its year, whether the athlete competed as an individual or part of a team (Regionals or the Games™), and their final ranking for each competition. All competition experience data represented the athletes’ official (i.e., the athlete registered for the competition and received an official rank) CF competition participation history from 2012 to 2019. Collected data was then further treated to better describe each athlete’s history. The highest (i.e., lowest value) and lowest (i.e., highest value) ranks ever earned were identified, while average rank and standard deviation (SD) of ranks were calculated, for athletes who made two or more appearances at any stage of the competition. Additional metrics used to describe experience at a given competition (i.e., CFO, regionals, Game™) included their total number of appearances, years since their first appearance, their participation rate in each event (i.e., total appearances divided by years since first appearance), and consecutive appearances prior to 2020. Descriptive characteristics of individual, team, and total history at each level of the competition are presented in Table 2.

**Table 2:** Athlete performance history in CrossFit® competition

	*n*	Individual experience	*n*	Team experience
*CrossFit® open*				
Highest rank	216	2017 ± 11,095 (2–142,355)		N/A
Lowest rank	216	24,257 ± 41,995 (35–216,285)		
Average rank	206	7857 ± 14,465 (20–93,754)		
SD of ranks	206	11,230 ± 21,722 (14–129,821)		
Appearances	220	4 ± 2 (0–8)		
Since first appearance (y)	220	4 ± 2 (0–7)		
Consecutive appearances	220	4 ± 2 (0–8)		
Participation rate (%)	216	92.4 ± 14.5 (20.0–100.0)		
*Regionals*				
Highest rank	70	17 ± 12 (1–48)	59	12 ± 8 (1–30)
Lowest rank	70	27 ± 14 (5–99)	59	17 ± 9 (2–35)
Average rank	39	19 ± 8 (3–35)	27	15 ± 6 (5–27)
SD of ranks	39	9 ± 9 (1–55)	27	8 ± 5 (1–24)
Appearances	220	1 ± 1 (0–6)	220	0 ± 1 (0–4)
Since first appearance (y)	70	1 ± 2 (0–5)	59	1 ± 1 (0–5)
Consecutive appearances	22	3 ± 1 (2–6)	6	2 ± 1 (2–4)
Participation rate (%)	53	66.2 ± 30.8 (17.0–100.0)	49	50.3 ± 23.9 (14.0–100.0)
*The Games™*				
Highest rank	19	35 ± 26 (2–84)	16	22 ± 13 (2–37)
Lowest rank	19	42 ± 21 (16–84)	16	22 ± 13 (2–37)
Average rank	9	22 ± 11 (6–39)	N/A	
SD of ranks	9	8 ± 3 (1–13)	N/A	
Appearances	220	0 ± 1 (0–5)	220	0 ± 0 (0–2)
Since first appearance (y)	19	2 ± 2 (0–5)	16	0 ± 1 (0–2)
Consecutive appearances	2	3 ± 1 (2–4)	N/A	
Participation rate (%)	11	59.6 ± 26.0 (20.0–100.0)	15	39.1 ± 14.0 (20.0–67.0)

### Statistical analyses

Data were modeled using both a frequentist and Bayesian approach. Prior to assessing relationships, results of the Shapiro-Wilks test indicated that most variables were not normally distributed. Therefore, the frequentist approach used the non-parametric, Kendall’s tau (τ) procedure to assess relationships between variables. The Bayesian approach then assessed the likelihood of observed relationships under the alternative hypothesis compared to the null hypothesis (i.e., no relationship between variables) by calculating Bayes factors (i.e., BF_10_) for each correlation using default prior scales [27]. These were interpreted according to the recommendations of Wagenmakers et al. [28] where a relationship was interpreted as evidence in favor of the null hypothesis when BF_10_ < 1. Otherwise, it was interpreted as “anecdotally” (1 < BF_10_ < 3), “moderately” (3 < BF_10_ < 10), “strongly” (10 < BF_10_ < 30), “very strongly” (30 < BF_10_ < 100), or “extremely” (BF_10_ > 100) in favor of the alternative hypothesis. All statistical analyses were performed using JASP 0.14.1.0 (Amsterdam, the Netherlands) with a criterion alpha set at *p* ≤ 0.05. All data are reported as mean ± standard deviation.

## Results

**Fig. 1:**
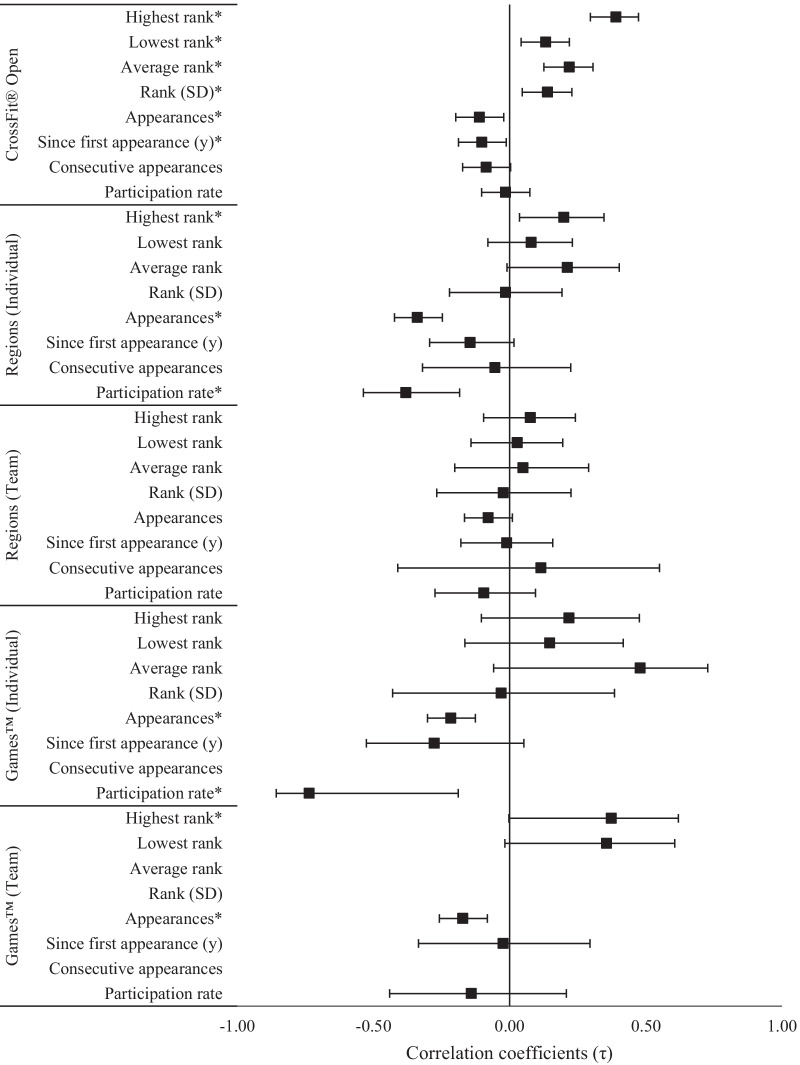
Relationships (± 95% CI) between competition experience and 2020 CFO Overall rank. *Significant (p < 0.05) relationship between ranks

The relationships between competition experience and 2020 CFO overall ranking are illustrated in Fig. 1. For overall rank in the 2020 CFO, evidence *extremely* (*p* < 0.001, BF_10_ > 100) favored positive relationships with the highest (and average) overall ranks earned across all previous CFO competitions. Evidence also *extremely* favored negative relationships with the total number of appearances (and participation rate since first appearance) at regional and Games™ competitions as an individual athlete (*p* < 0.001, BF_10_ > 100), as well as the number of appearances as a member of a team at the Games™ (*p* = 0.002, BF_10_ > 100). There was *strong* evidence favoring a negative relationship with an athlete’s individual participation rate at the Games™ (*p* = 0.002, BF_10_ = 29.8), and *moderate* evidence for the athlete’s lowest CFO rank (*p* = 0.004, BF_10_ = 5.7) and SD in CFO rankings (*p* = 0.004, BF_10_ = 7.3) to be positively related to 2020 CFO rank. Evidence for all other relationships was either *anecdotal* or favored the null hypothesis.

**Fig. 2:**
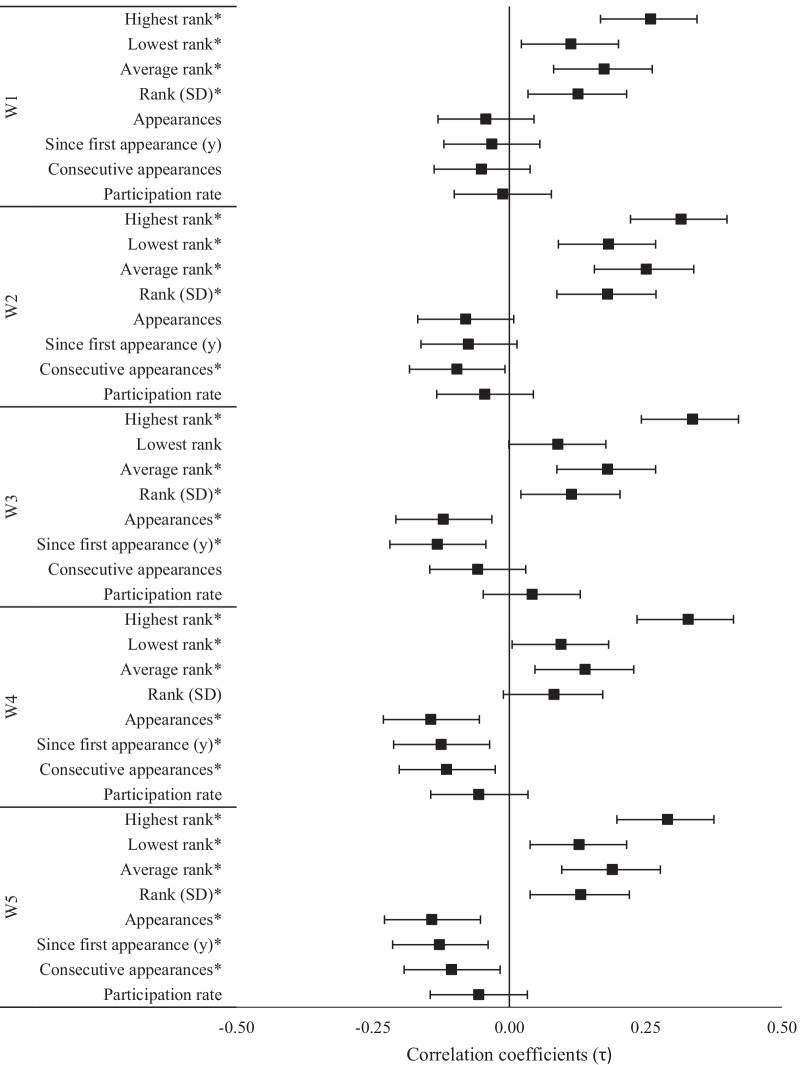
Relationships (± 95% CI) between past CFO experiences and 2020 CFO workout ranks. *Significant (*p* < 0.05) relationship between ranks

**Fig. 3:**
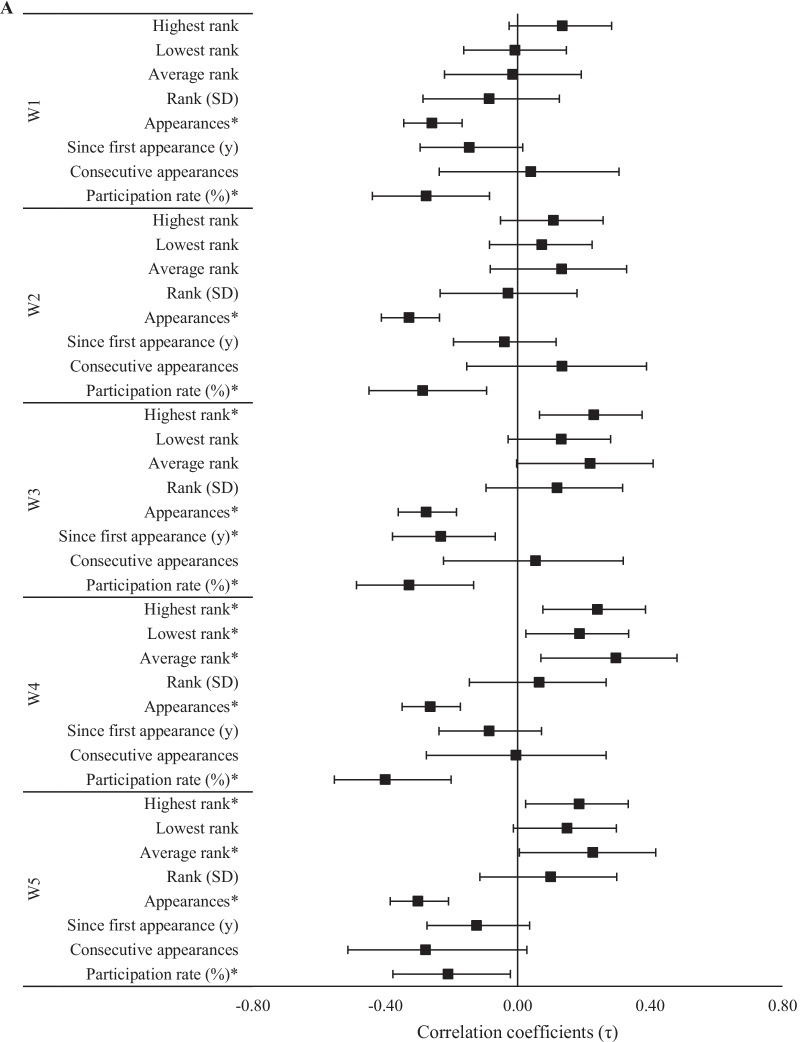

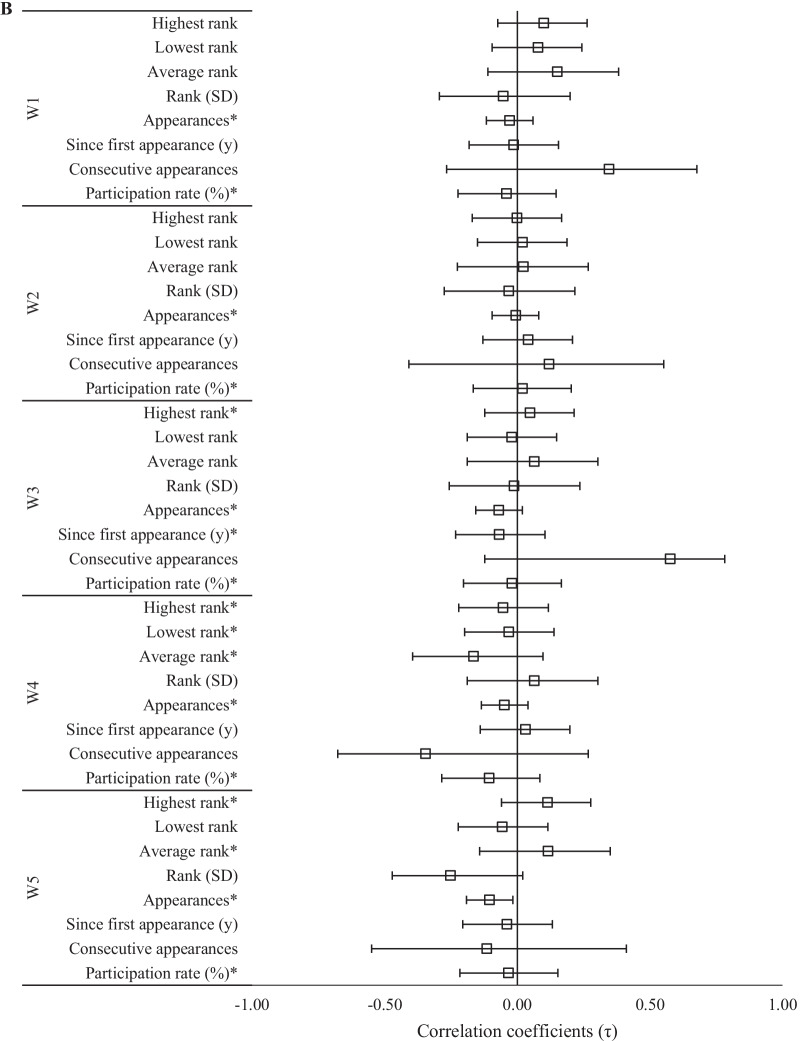
Relationships (± 95% CI) between 2020 CFO workout ranks and past experiences as **A** an individual and **B** team regional athlete. *Significant (*p* < 0.05) relationship between ranks

**Fig. 4:**
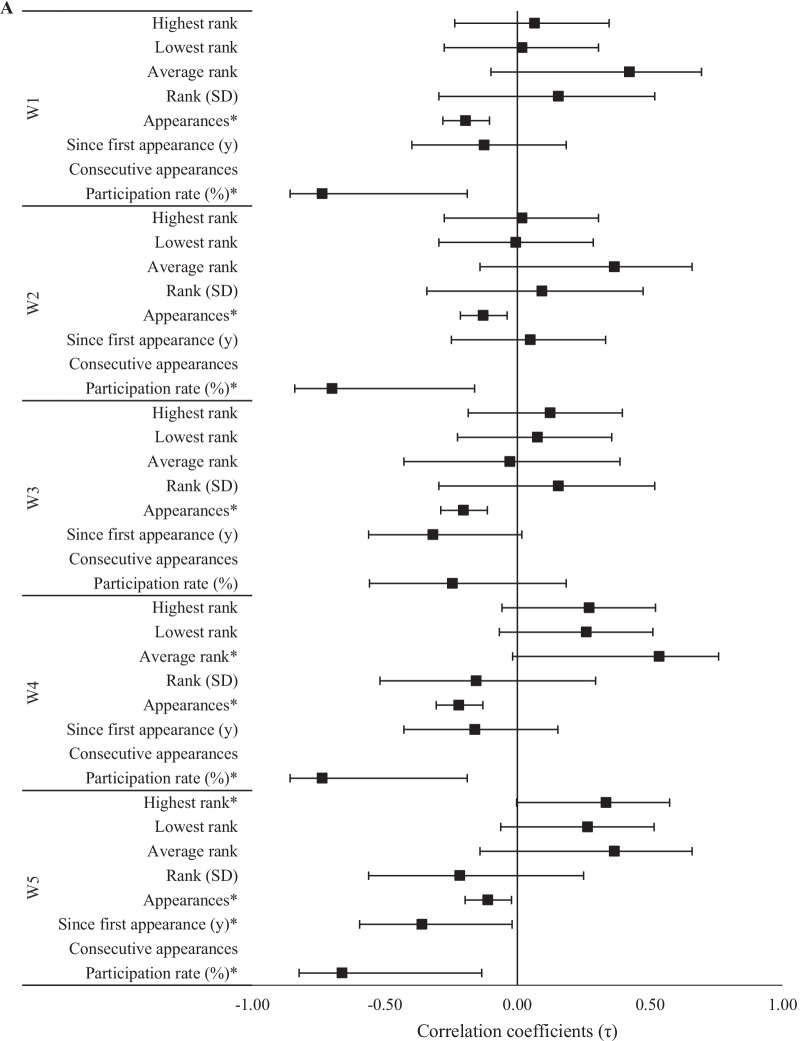

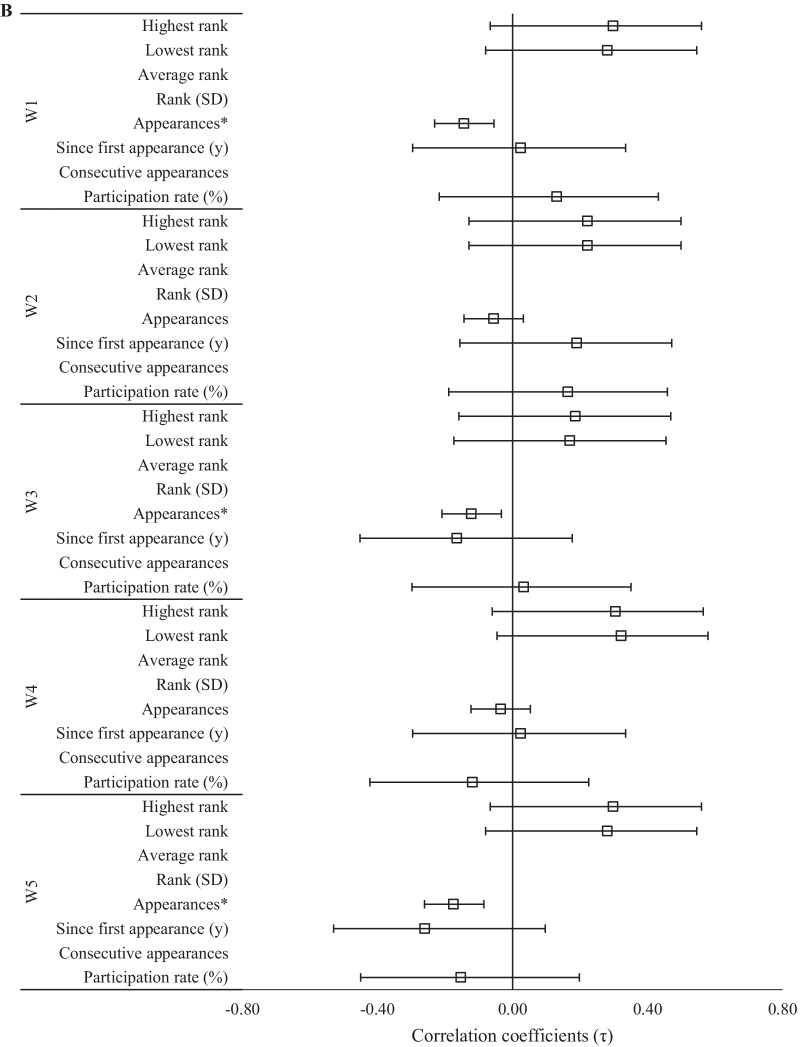
Relationships (± 95% CI) between 2020 CFO workout ranks and past experiences as **A** an individual and **B** team Games™ athlete. *Significant (*p* < 0.05) relationship between ranks

The calculated correlation coefficients between competitive experience variables and the athletes’ rank and score on each workout of the 2020 CFO were nearly identical. Observed relationships only differed by sign when a better score on a specific workout was negatively related to rank. Relationships between competitive experience variables and ranking in each 2020 workouts are illustrated in Figs. 2, 3 and 4.

Evidence for the highest CFO rank being positively related to 2020 CFO workout ranks was *extreme* (τ = 0.26–0.34, *p* < 0.001, BF_10_ > 100). Average CFO rank was also positively related to ranks earned in each workout (τ = 0.14–0.25, *p* < 0.003), but evidence was *extreme* for W2, W3, and W5, *very strong* for W1 (BF_10_ = 87.6), and moderate for W4 (BF_10_ = 7.5). Workout ranking was also related to the lowest CFO rank (τ = 0.10–0.18, *p* < 0.05) and SD of CFO ranks (τ = 0.11–0.18, *p* < 0.05) except for W3 and W4, respectively. However, evidence for these relationships was only *extreme* for W2 and *moderate* for W5 (BF_10_ = 4.4–4.5). Likewise, the SD of CFO ranks was positively related to performance in each workout (τ = 0.11–0.18, *p* < 0.05) except for W4, and evidence was only *extreme* for W2 and *moderate* for W5 (BF_10_ = 4.4). Evidence *strongly* favored negative relationships (τ = − 0.14, *p* = 0.003, BF_10_ = 4.0–4.6) between total CFO appearances and W4 and W5 rankings, and *moderately* favored W3 rank (τ = − 0.12, *p* = 0.013, BF_10_ = 3.1). Evidence also *moderately* favored negative relationships between the time (in years) since the athletes’ first CFO competition and W3–W5 ranks (τ = − 0.13, *p* < 0.05, BF_10_ = 4.0–6.0). Otherwise, evidence for all other relationships involving previous CFO experience was either *anecdotal* or favored the null hypothesis.

The magnitude of relationships between 2020 CFO workout ranks and experience at regional competitions was dependent upon whether the athlete competed as an individual or as part of a team. Regarding individual competition, evidence *extremely* favored negative relationships between the number of appearances at regional competitions and rank in each workout (τ = − 0.26 to − 0.33, *p* < 0.001), and between participation rate and W4 (τ = − 0.40, *p* < 0.001). Negative relationships between participation rate and W3 rank (τ = − 0.33, *p* < 0.001, BF_10_ = 63.2), as well as W1 and W2 ranks (τ = − 0.28 to − 0.29, *p* < 0.05, BF_10_ = 11.6–15.9), were supported by *very strong* and *strong* evidence, respectively. Positive relationships between W4 rank and the highest and average regional competition ranks were supported by *strong* (τ = 0.24, *p* = 0.004, BF_10_ = 11.2) and *moderate* (τ = 0.30, *p* = 0.009, BF_10_ = 6.5) evidence, respectively. Meanwhile, for W3, *moderate* evidence supported a positive relationship to highest regional competition rank (τ = 0.23, *p* = 0.005, BF_10_ = 7.8) and a negative relationship to the amount of time (in years) since an athlete’s first individual regional appearance (τ = − 0.23, *p* = 0.010, BF_10_ = 8.3). All other relationships involving previous regional competition experience, including as a member of a team, were either *anecdotal* or favored the null hypothesis.

Relationships between 2020 CFO workout ranks and experiences at the Games™ were also dependent upon whether the athlete competed as an individual or as part of a team. *Extreme* evidence supported negative relationships between the number of individual appearances and ranking in W1, W3, and W4 (τ = − 0.20 to − 0.23, *p* < 0.001), and between the number of appearances on a team and W5 rank (τ = − 0.18, *p* = 0.002). *Strong* evidence supported negative relationships between individual participation rate and ranks in each workout (τ = − 0.66 to − 0.74, *p* < 0.05, BF_10_ = 13.0–29.8) except for W3, and between the of appearances as a team member and W1 rank (τ = − 0.14, *p* = 0.009, BF_10_ = 13.9). There was also moderate evidence for team appearances and W3 rank (τ = − 0.12, *p* = 0.027, BF_10_ = 3.3). All other relationships involving Games™ experience were either *anecdotal* or favored the null hypothesis.

## Discussion

This study examined relationships between prior CF competition experiences and performance in the 2020 CFO among the top 1000-ranked athletes. Previously, years of CF experience [6, 9] and past ranks at various stages of the Games™ competition [6] were found to be related, albeit variably, to CF performance. Our data support and expand upon those findings by documenting relationships between 2020 CFO performance and past competition experiences distinguished by appearances, competition stage, and division rank. Specifically, positive relationships were observed between all metrics of CFO rank (i.e., highest, lowest, average, and SD) and 2020 CFO rank (overall and for nearly all workouts). With only two exceptions, rank (overall and for each workout) was also negatively related to the number of appearances at each stage in previous years (i.e., more appearances at each stage were associated with higher ranks [lower numerical value]). Participation rate as an individual regional and Games™ competitor, was also negatively related to most workouts, and at the Games™, was more important than final placement. Few relationships were observed, however, when the athlete’s appearance at regional or Games™ competition was by way of team. Still, more associations were seen when the athlete was part of a team that competed at the Games™ compared to those that never advanced beyond the regional level. Although preliminary, these data may be useful for determining when more specific sample descriptions are needed in CF research. Further, this line of research may help athletes and coaches distinguish the importance of various experiences versus physiological characteristics to performance at different levels of CF competition.

With only two exceptions (i.e., lowest CFO rank and W3; SD of ranks and W4), better CFO performance history was most consistently linked with better 2020 performance. Performance (overall and in each workout) was *extremely* favored to be related to the highest rank achieved in previous CFO competitions, as well as to the number of times an athlete appeared as an individual, regional competitor. Indeed, when the competition included a regional stage, only top-ranked competitors within the 17 worldwide regions (i.e., 10th–30th depending on the region or the top 0.01% worldwide) would progress beyond the CFO. The number of individual appearances at the Games™ was also related to ranking (overall and in each workout), but evidence was not as strong and likely the consequence of far fewer athletes in this study ever reaching this final stage. Regardless, our data supports evidence of past CFO ranking being a predictor (*r*^2^ = 0.28–0.59) of performance in future competitions [6]. In fact, there is only a single instance between previous work [6] and the present study where a significant relationship was not observed between the athlete’s personal best and current CFO performances. That instance occurred in 2018 when competitors had to perform a maximal power clean immediately after another workout; a task that was better predicted by muscle size, strength, power, and resistance training experience. However, CF workouts are not commonly scored by maximum weight lifted. It is more common for specific loads and repetition schemes to be assigned to some combination of exercises, which are then either scored as the number of repetitions completed within a time limit or by how quickly the athlete completes the assigned work [5, 25, 29]. While the present data does not support or refute the importance of the athlete’s physiological attributes, those who advance and face better competition more frequently appear to have a tactical advantage. Those with sufficient experience [21–24], particularly when it was gained against better competition [18], are more likely to recognize familiar elements in novel workouts and devise a better strategy to manage fatigue and optimize performance.

Interestingly, few relationships were seen between historical rank in later stages of the competition and 2020 CFO performance. Despite *extreme* evidence predominantly favoring relationships with appearances (at regions and Games™), evidence for ranking history at the regional stage was generally *anecdotal*-to-*moderate* and even less convincing for Games™ rankings. The reasons for this are currently unclear but a few potential explanations warrant further investigation. First, CFO competitors and those who advance may represent two different populations. Serafini et al. [8] found differences in measures of strength, power, and sport-specific skill among quintiles created from the top 1500 athletes of the 2016 CFO. Although these differences predominantly favored the top-ranked quintile compared to all others, the accuracy and timing of the self-reported data (obtained from the athletes’ online profile) was unknown. Nevertheless, those findings were corroborated by Mangine and colleagues [30], who noted lower body fat percentage and greater bone and non-bone lean mass, muscle morphology characteristics, isometric strength, peak aerobic capacity, and 3-min “all-out” cycling performance in an advanced group of athletes that possessed regional and Games™ experience compared to those who had never progressed beyond the CFO. Still, the advanced group was mainly comprised of team athletes, and differences between experienced team and individual athletes have yet to be examined. Another explanation could be that CFO workouts are not comparable to those that have appeared at regional events and the Games™. Even though any workout might appear at any stage of the competition, later-stage workouts often incorporate higher loads for a given repetition scheme, more repetitions at a given load, and/or components (e.g., running, obstacle courses, rope climbing, and peg board ascents) that require more skill, specific equipment, or are too difficult to standardize in the CFO [25, 29]. Additionally, the competition structure between the CFO and regional or Games™ events are vastly different. For instance, participants of the CFO may perform any given event as many times as they wish within the allotted timeframe. This may allow for attempting different pacing strategies, transitions, and general approaches to the workout to optimize their performance. With this, participants of the CFO are also given an entire week between competitive events, whereas, Regional and Games™ participants often perform multiple events in a single day and repeat this over several consecutive days. Currently, however, there are no established methods for quantifying difficulty or making fair comparisons amongst all the potential design variations of CF workouts. Until such methods exist, any stated difficulty differences are speculative at best and highly subjective to personal bias. Finally, the lack of relationships may have simply been the consequence of reduced statistical power. Of the athletes who competed in the 2020 CFO, most had CFO experience (*n* = 216) but far fewer possessed at least one year of either individual or team regional (*n* = 104) or Games™ (*n* = 32) experience. Athletes who had never advanced beyond the CFO still received a score of zero for appearances (at regions and the Games™), but because no value could be assigned for rank, their cases were not considered when examining the relationships between ranks at each stage. Thus, for the time being, it appears that possessing the skill to advance beyond the CFO is more meaningful to future performance than one’s eventual rank in later competition stages.

Team regional and Games™ experience was less valuable than individual competition experience. Appearances as a regional team athlete was only related to W5 performance, whereas the number of times an athlete progressed to the Games™ as part of a team was only slightly more advantageous. Though many of the reasons previously cited for historical rank may also be relevant here, the most likely explanation is that CFO performance is not the sole consideration for team composition. There are several unique competition aspects that must be considered when forming a team. For instance, team composition rules have typically required an equal number of men and women, who are only eligible if they: (1) trained at the same location for more than half of the year, (2) stated this affiliation during the registration process, and (3) participated in at least one CFO workout [31]. Because of these stipulations, it is possible for a team to not be comprised of the highest-ranking individuals from a given location. Furthermore, team competition workouts emphasize the concept of “teamwork” by often incorporating elements that cannot be performed individually (e.g., synchronized movements, relays, the “worm”) [25, 29]. Drastic differences in individual body size, skill, and physiology may all negatively impact the team’s ability to function as a unit and perform these tasks effectively. Consequently, team leadership may select athletes based on their shared similarities or how well they complement each other’s strengths and weaknesses. Finally, those who qualify for advancement as both an individual and team athlete may simply decide not to continue in both competitions. In any case, this appears to be the first study to distinguish between aspects relevant to individual and team CF performance. Further research is needed to better clarify the differences, if any exist, between these two types of athletes at various stages of the competition.

The findings of this study suggest that 2020 CFO performance was modulated by an athlete’s past competition experiences. However, limitations to this study have left several areas in need of further investigation. These seem to be predominantly related to our definition of success (i.e., performance in the 2020 CFO competition). The fixed nature of this selection limits the generalizability of our findings over multiple years. Since different workouts comprise the CFO each year [32], the relevance of specific traits may also change annually. Future studies might overcome this limitation by examining the consistency of our observations across multiple CFO competitions. Moreover, success may be better described by extending this strategy to the truest metric of accomplishment in this sport: performance at the Games™. Although this might be perceived as a limitation because the sample would be even more focused than the one used in this study (i.e., the top 1000 equated to the top 0.7%) [19], doing so would ensure that all relevant stages of the competition were sufficiently represented. Additionally, there is a degree of uncertainty about the accuracy of CFO scores. Scores are self-reported and competition officials must rely on local judges and video submissions to verify their accuracy [31]. With hundreds of thousands of competitor scores needing verification within a 4-day window each year [19], the possibility of miscounted repetitions, technical standard violations, and blatant cheating going unnoticed is ever present. Randomizing the sample selection process helps to minimize the influence of these inaccuracies, but it cannot remove them. Only a multi-site, longitudinal study where workouts are completed in the presence of research team members could overcome this limitation.

## Conclusions

An athlete’s competitive experience is a contextual lens that alters the relevance of scientific findings. Studies often limit their descriptions of experience to the athlete’s competition level, years playing the sport, or at minimum, a list of notable achievements. The results of this pilot study suggest that more detail is needed to clearly examine factors related to CF performance. Here, performance was defined by 2020 CFO ranking (or workout score—both produced similar results), and it was most consistently linked to performance in past CFO competitions. Greater success, identified by the athlete’s CFO rank history or appearances as an individual regional or Games™ competitor, was associated with a higher 2020 CFO rank (overall and in each workout). Athletes who more frequently and consistently advanced to the later competition stages ranked higher and performed better in the 2020 CFO. However, simply appearing in later competition stages was not an automatic indicator of CFO success. Relationships were influenced by whether the athlete had advanced as an individual or team competitor, and potentially by the circumstances that facilitated their advancement. Being part of a regional team was only relevant to one 2020 workout, whereas team experience at the Games™ was a more consistent indicator. Future studies should corroborate these findings across multiple years of CFO competition, as well as in combination with relevant physiological characteristics. A potential caveat also worth further investigation may have to do with the rationale for the athlete’s presence on the team (i.e., based on athletic ability, team needs, or both). Finally, the present study limited its examination to CF competition experience. While no other sport can adequately be likened to CF, an athlete’s overall sports experience, any resultant traits and skills, and their overall motivations and perceptions towards sports, may all influence their performance in CF competitions and are worth investigating.

## Data Availability

The data used for this manuscript is publicly available within an online database [25]. Please contact the author to request the random sample generated for this study.

## Abbreviations

AMRAP: As many repetitions as possible
BF: Bayes factor
CF: CrossFit®
CFO: CrossFit® open
PDT: Pacific daylight time
SD: Standard deviation
W1–W5: Workouts 1–5
TTC: Time-to-completion
